# Epidemiologic Evaluation of Human Papillomavirus Type Competition and the Potential for Type Replacement Post-Vaccination

**DOI:** 10.1371/journal.pone.0166329

**Published:** 2016-12-22

**Authors:** Joseph E. Tota, Mengzhu Jiang, Agnihotram V. Ramanakumar, Stephen D. Walter, Jay S. Kaufman, François Coutlée, Harriet Richardson, Ann N. Burchell, Anita Koushik, Marie Hélène Mayrand, Luisa L. Villa, Eduardo L. Franco

**Affiliations:** 1 McGill University, Department of Oncology, Montreal, Québec, Canada; 2 McGill University, Department of Epidemiology, Biostatistics, and Occupational Health, Montreal, Québec, Canada; 3 National Cancer Institute, Division of Cancer Epidemiology and Genetics, Infections and Immunoepidemiology Branch, Rockville, Maryland, United States of America; 4 McMaster University, Department of Clinical Epidemiology and Biostatistics, Hamilton, Ontario, Canada; 5 Université de Montréal, Département de Microbiologie et Infectiologie, Montreal, Québec, Canada; 6 Université de Montréal Hospital Research Centre, Montreal, Québec, Canada; 7 Queen’s University, Department of Public Health Sciences, Kingston, Ontario, Canada; 8 St. Michael’s Hospital, Department of Family and Community Medicine and Centre for Research on Inner City Health, Li Ka Shing Knowledge Institute, Toronto, Ontario, Canada; 9 Université de Montréal, Département de médecine sociale et préventive, Montreal, Québec, Canada; 10 Université de Montréal, Département d’obstétrique-gynécologie et Médecine Sociale et Préventive, Montreal, Québec, Canada; 11 Universidade de São Paulo, Department of Radiology and Oncology, School of Medicine, São Paulo, Brazil; Penn State University School of Medicine, UNITED STATES

## Abstract

**Background:**

Millions of women have been vaccinated with one of two first-generation human papillomavirus (HPV) vaccines. Both vaccines remain in use and target two oncogenic types (HPVs 16 and 18); however, if these types naturally compete with others that are not targeted, type replacement may occur following reductions in the circulating prevalence of targeted types. To explore the potential for type replacement, we evaluated natural HPV type competition in unvaccinated females.

**Methods:**

Valid HPV DNA typing information was available from five epidemiological studies conducted in Canada and Brazil (n = 14,685; enrollment across studies took place between1993 and 2010), which used similar consensus-primer PCR assays, capable of detecting up to 40 HPV types. A total of 38,088 cervicovaginal specimens were available for inclusion in our analyses evaluating HPV type-type interactions involving vaccine-targeted types (6, 11, 16, and 18), and infection with each of the other HPV types.

**Results:**

Across the studies, the average age of participants ranged from 21.0 to 43.7 years. HPV16 was the most common type (prevalence range: 1.0% to 13.8%), and in general HPV types were more likely to be detected as part of a multiple infection than as single infections. In our analyses focusing on each of the vaccine-targeted HPV types separately, many significant positive associations were observed (particularly involving HPV16); however, we did not observe any statistically significant negative associations.

**Conclusions:**

Our findings suggest that natural HPV type competition does not exist, and that type replacement is unlikely to occur in vaccinated populations.

## Introduction

Infection with high-oncogenic risk human papillomavirus (HR-HPV) is a necessary cause of cervical cancer in women [1] and an important cause of other anogenital cancers in both genders [2]. In addition, some low-oncogenic risk (LR) HPV infections may cause benign lesions known as acuminate condylomata (genital warts), as well as low grade squamous intraepithelial cervical lesions. Two highly effective HPV vaccines have been administered to millions of women around the world (Merck’s Gardasil© and GlaxoSmithKline's Cervarix©) [3, 4], offering protection against two HR-HPV types (16 and 18)–responsible for approximately 70% of cervical cancer cases. Only Gardasil protects against additional LR-HPV types (6 and 11) that cause approximately 90% of genital warts cases [5–7]. Although HPV vaccination is eventually expected to reduce the burden of disease attributable to these HPV types, there is concern that it may lead to “type replacement” [8], i.e., an increase in the prevalence of other non-vaccine HPV types following the reduction of vaccine-targeted types [9, 10].

For type replacement to occur, a biological prerequisite is that different HPV types must compete with one another for niche occupation during natural infection [9–11]. We recently described different epidemiological approaches to evaluate HPV type competition in order to gain insight regarding the likelihood of type replacement [10]. The two main approaches include construction of Kaplan-Meier curves and Cox models to evaluate sequential acquisition and clearance of HPV types according to HPV status with vaccine-targeted types; and construction of logistic regression models for each vaccine-targeted type to explore whether infection with these types may be associated with infection by other HPV types. A number of cohort studies evaluating the natural history of HPV infections among females have suggested that those infected with HPV (any type) are generally at higher risk of acquiring other types [12–15], or at about equal risk of acquiring and clearing existing infections [12–17]. Similarly, other recent cross-sectional studies that have investigated clustering patterns of different HPV types have found that females infected with HPV (vaccine or other types) are more likely to be infected with additional HPV types [18–26]. These previous studies reported very few negative associations, therefore providing some reassurance that type competition does not exist and that replacement is unlikely. Despite the large sample size of some of these studies, few or no co-infections were observed for rare HPV types, leading to non-positivity or low precision for some comparisons. In addition, evaluation of pairwise interactions in these studies did not account for presence of other HPV types, which may have introduced some confounding [10].

To evaluate HPV type competition in the current study, we applied a hierarchical (Bayesian) regression approach that employs shrinkage and adjustment for confounders, as well as other HPV types. Data were available from five pre-vaccination studies conducted among females in Canada and Brazil.

## Methods

### Study population and design

Participant data for the current analysis came from five studies conducted by the Division of Cancer Epidemiology, McGill University. They included: a) the Ludwig-McGill cohort study (São Paulo, Brazil; n = 2462) [27], b) the HPV Infection and Transmission among Couples through Heterosexual activity (HITCH) cohort study (Montreal, Canada; n = 1038; 502 females, 536 males) [28], c) the McGill-Concordia cohort study (Montreal, Canada; n = 636) [29], d) the Biomarkers of Cervical Cancer Risk (BCCR) case-control study (Montreal, Canada; n = 1687) [30], and e) the Canadian Cervical Cancer Screening Trial (CCCaST) (Montreal/St. John’s, Canada; n = 10,154) [31]. Recruitment for these studies took place between 1993 (Ludwig-McGill) and 2010 (HITCH), and age of participants ranged from 18 (Ludwig-McGill, HITCH and McGill-Concordia) to 69 years (CCCaST). Protocols for each of the five studies have been described in detail elsewhere [27–31]. Briefly, the three cohort studies (Ludwig-McGill, HITCH, and McGill-Concordia) were designed to evaluate the natural history of HPV infection among females, and transmission of HPV among heterosexual couples (male data from the HITCH study was not included in the current analysis). BCCR is a case-control study that was originally designed to evaluate the role of biomarkers in the etiology of cervical precancer and cancer, and CCCaST was the first North American randomized controlled trial to compare Pap cytology versus HPV testing in screening for cervical cancer. Subjects completed questionnaires to collect information on important demographic and lifestyle variables; and provided cervical samples (self or provider collected) for HPV testing at each of their clinic visits. All participants provided written informed consent and each study was approved by review boards or ethical committees at McGill University and other participating institutions.

### HPV DNA detection and genotyping

In the three cohort studies, cervical specimens were collected and tested for HPV at each clinic visit (every four months during the first year of follow-up/twice annually in subsequent years of follow-up in the Ludwig-McGill and HITCH studies; and twice annually in the McGill-Concordia study). Subjects from the Ludwig-McGill, HITCH, and McGill-Concordia studies contributed an average of 9.0, 4.4, and 4.2 cervical specimens for HPV testing, respectively; whereas subjects from the BCCR and CCCaST studies contributed only one specimen for HPV testing.

Details regarding specific sample collection and HPV testing protocols for each study have been described in detail elsewhere [27–31]. Briefly, all studies employed consensus primer PCR assays (L1 PGMY or MY09/11 and hybridization with oligonucleotide probes and restriction fragment length polymorphism analysis, line blot assay, or linear array), which are capable of detecting between 27 and 40 different HPV types. The MY09/11 and PGMY09/11 protocols are both very sensitive with good overall agreement (kappa range = 0.68–0.83) [32–34] and modifications to the MY09/11 protocol (leading to the PGMY09/11 protocol) has resulted in even greater test sensitivity [32]. Although the genotyping procedure in the Ludwig-McGill study (hybridization with individual oligonucleotide probes and restriction fragment-length polymorphism analysis) did not allow us to distinguish between vaccine-targeted HPV types 6 and 11, these are two of the most closely related HPV types (with similar biological and pathological properties) [35], therefore grouping them was not viewed as a major limitation. Nonetheless, we evaluated HPVs 6 and 11 together, as well as separately in the other four studies. Since types that are phylogenetically related (i.e., from the same species) share a large proportion of their nucleotide sequence (≥60%) and display similar biological properties, we suspected that types from the same species would be more likely to compete [35, 36]. HPV types belonging to the same species as HPV6/11 (α-10) include 13, 44, and 74; as HPV16 (α-9) include 31, 33, 35, 52, 58, and 67; and as HPV18 (α-7) include 39, 45, 59, 68, and 70.

### Statistical analysis

We investigated the association between infection with the vaccine preventable types and infection with each of the other HPV types using pooled data from the five studies. Bayesian hierarchical regression models were constructed for vaccine preventable types 6, 11 (6/11 combined), 16, and 18. Age and lifetime number of sex partners were chosen as covariates a priori, since they are strong predictors of HPV infection [2]. Thus the primary analyses excluded a portion of CCCaST participants who were missing baseline data on lifetime number sex partners. Models for 6/11 combined, 16, and 18 included data from all five studies. Models for 6 and 11 separately excluded the Ludwig-McGill study, as explained above. Secondary analyses included the CCCaST participants with missing information on lifetime number of sex partners by excluding it as a covariate. We also conducted analyses for each study separately.

Specifically, the probability of infection with the vaccine preventable type was modeled in a 2-tier hierarchical model, where subjects’ study visits were nested within subjects in order to account for subject-level clustering. At the visit level, a logistic model was fitted with infection with the vaccine preventable type as the outcome and every other HPV type and age at the time of the visit as predictors. At the subject level, the subject-specific intercepts were modeled by accounting for lifetime number of sex partners at baseline, as well as the study that the subject came from for the pooled models. Thus, the odds ratio (OR) estimate for each HPV type represents the odds of detection of the vaccine-preventable type in the presence of that HPV type compared to the odds of detection of the vaccine-preventable type in the absence of that particular HPV type, adjusted for all other HPV types, age at visit, lifetime number of sex partners at baseline, and study.

In order to improve the precision of the estimates for the effect of the presence of other HPV types on the presence of the vaccine preventable type, the logistic regression parameters for all the other HPV types were assumed to be normally distributed around an overall mean effect of co-infection. Diffuse or wide prior distributions were used for all other parameters. All analyses were conducted using WinBUGS software version 1.4.3 (MRC Biostatistics Unit, Cambridge).

The additional hierarchical component on the coefficients of other HPV types produces a shrinkage effect, whereby unstable estimates with large variances are drawn closer to the mean. The assumption introduces a bias in favour of reducing variance and potentially reducing mean squared error [37]. To explore the possible effect of this bias, we also compared our results with estimates for HPV type associations calculated using the maximum likelihood method.

## Results

Subject characteristics stratified by study population are listed in Table 1. The average age of participants at enrollment across the five studies ranged from 21.0 (HITCH study) to 43.7 years (CCCaST study). Given that they were studies of young adult women, HITCH and McGill-Concordia studies included few females that were married/common-law (14.1% and 18.0%, respectively) or that had ever been pregnant (9.8% and 16.2%, respectively). Compared with subjects from the four Canadian studies, Brazilian Ludwig-McGill study participants reported fewer lifetime sexual partners (87% had less than five partners) and the majority rarely used condoms (less than 4% used condoms regularly). Most subjects in the McGill-Concordia, HITCH and BCCR studies indicated that they were never smokers (62.7%, 62.3% and 50.0%, respectively); whereas the majority of Ludwig-McGill and CCCaST participants reported that they were current/former smokers (52.5% and 79.8%, respectively).

**Table 1:** Characteristics of female participants at baseline/enrollment in five epidemiological studies.

Characteristic	Ludwig-McGill	McGill-Concordia	HITCH	CCCaST ^a^	BCCR
	n = 2462	n = 636	n = 502	n = 10154	n = 985
	n (%)	n (%)	n (%)	n (%)	n (%)
**Age**, years, mean (SD)	32.7 (8.8)	22.5 (4.0)	21.0 (2.1)	43.7 (9.1)	30.1(9.8)
**Marital status**					
Single	252 (10.2)	495 (77.8)	425 (84.7)	1262 (12.4)	450 (45.7)
Married/common law	2011 (81.7)	114 (18.0)	71 (14.1)	7441 (73.3)	474 (48.2)
Widowed/divorced	197 (8.0)	14 (2.2)	6 (1.2)	1353 (13.3)	57 (5.8)
Missing	2 (0.1)	13 (2.0)	0 (0.0)	98 (1.0)	4 (0.4)
**Age at sexual debut**					
< 16	479 (19.5)	125 (19.6)	45 (24.3)	557 (12.7)	243 (24.7)
≥ 16	1958 (79.5)	443 (69.7)	454 (75.1)	3795 (86.2)	702 (71.3)
Missing	25 (1.0)	68 (10.7)	3 (0.6)	48 (1.1)	40 (4.0)
**Lifetime # of sex partners**					
0–1	1089 (44.2)	135 (22.2)	54 (10.7)	851 (19.3)	163 (16.5)
4-Feb	1053 (42.8)	198 (32.1)	145 (28.9)	1251 (28.4)	291 (29.5)
≥ 5	318 (12.9)	277 (43.6)	303 (60.4)	2236 (50.8)	516 (52.4)
Missing	2 (0.1)	26 (4.1)	0 (0.0)	62 (1.4)	15 (1.5)
**# of pregnancies**					
0	47 (1.9)	511 (80.3)	452 (90.0)	806 (18.3)	471 (47.8)
2-Jan	894 (36.3)	97 (15.2)	47 (9.4)	2113 (48.0)	335 (34.0)
≥ 3	1502 (61.0)	6 (1.0)	2 (0.4)	1420 (32.3)	174 (17.7)
Missing	19 (0.8)	22 (3.5)	1 (0.2)	61 (1.4)	5 (0.5)
**Oral contraceptive use**					
Never	397 (16.1)	135 (21.2)	80 (16.0)	3958 (39.0)	91 (9.2)
Ever	2064 (83.9)	461 (72.5)	421 (83.9)	1496 (14.7) ^b^	882 (89.5)
Missing	1 (0.0)	40 (6.3)	1 (0.4)	4700 (46.3)	12 (1.2)
**Condom use**					
Never	936 (38.0)	30 (4.7)	16 (3.2)	4206 (41.4)	93 (9.4)
Rarely or sometimes	1398 (56.8)	209 (32.9)	185 (37.0)	1187 (11.7) ^b^	344 (34.9)
Regularly or always	92 (3.7)	362 (56.9)	300 (59.6)		536 (54.4)
Missing	36 (1.5)	35 (5.5)	1 (0.2)	4761 (46.9)	12 (1.2)
**Cigarette smoking**					
Never smoker	1168 (47.5)	399 (62.7)	313 (62.3)	1967 (19.4)	492 (50.0)
Former smoker	429 (17.4)	124 (19.5)	129 (25.7)	4928 (48.5)	189 (19.2)
Current smoker	864 (35.1)	99 (15.6)	60 (12.0)	3182 (31.3)	300 (30.4)

^a^ St. John’s study site (*n* = 5754) did not collect information on age at sexual debut, number of lifetime sex partners, or number of pregnancies. For these variables, percentage missing was based on the number of Montreal study site subjects only (*n* = 4400).

^b^ Checklist was used in CCCaST study only to evaluate whether subjects “ever” used oral contraceptives or condoms, along with other contraceptive methods.

**Fig 1:**
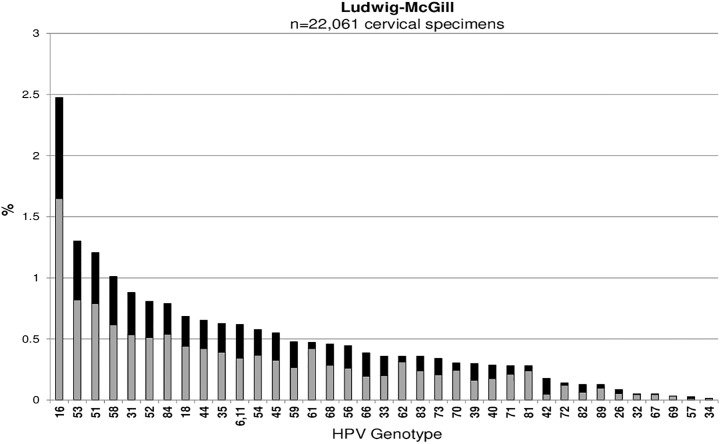
Human papillomavirus (HPV) genotype distribution of single (in light grey) and multiple infections (in black) in order of descending frequency in the Ludwig-McGill cohort study.

**Fig 2:**
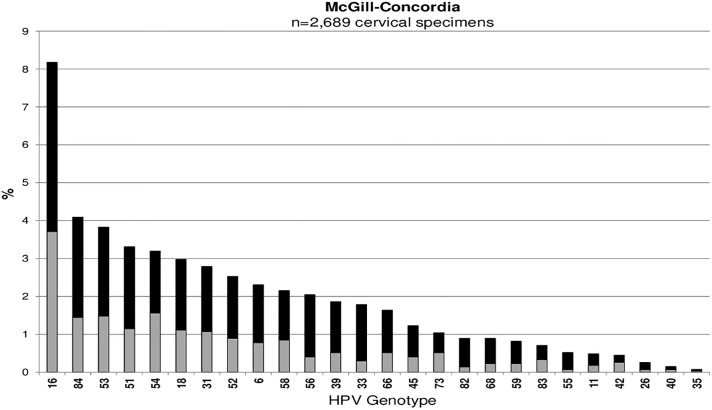
Human papillomavirus (HPV) genotype distribution of single (in light grey) and multiple infections (in black) in order of descending frequency in the McGill-Concordia cohort study.

**Fig 3:**
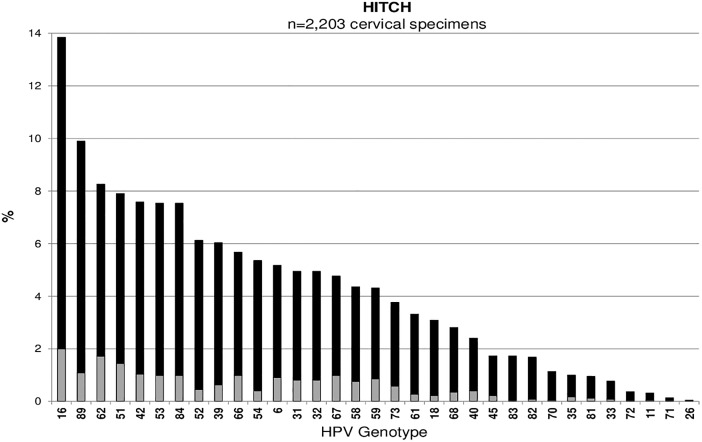
Human papillomavirus (HPV) genotype distribution of single (in light grey) and multiple infections (in black) in order of descending frequency in the HITCH cohort study.

**Fig 4:**
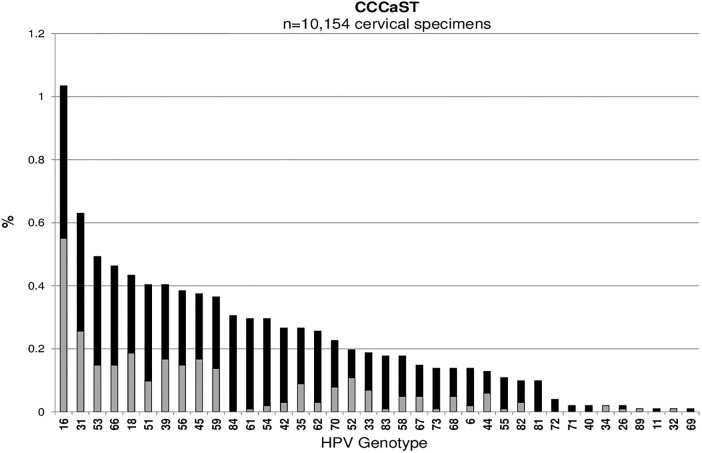
Human papillomavirus (HPV) genotype distribution of single (in light grey) and multiple infections (in black) in order of descending frequency in the CCCaST study.

**Fig 5:**
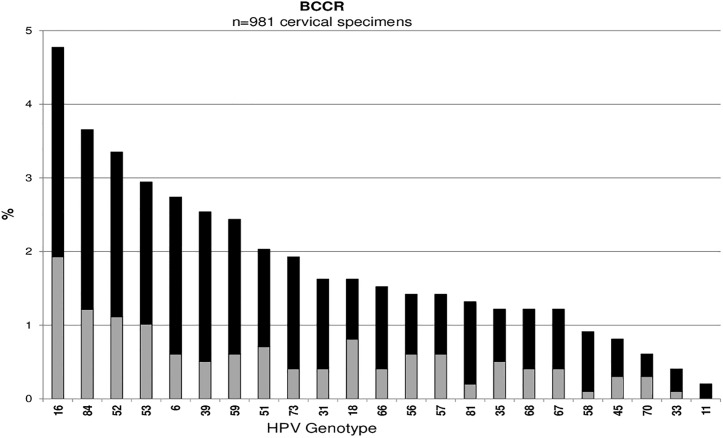
Human papillomavirus (HPV) genotype distribution of single (in light grey) and multiple infections (in black) in order of descending frequency in the BCCR case-control study.

Across all studies, HPV16 was the most common type detected among cervicovaginal specimens: Ludwig-McGill (n = 546, 2.5%), McGill-Concordia (n = 220, 8.2%), HITCH (n = 305, 13.8%), CCCaST (n = 105, 1.0%), and BCCR (n = 47, 4.8%) (Figs 1, 2, 3, 4 and 5). Although the ranking of other common HPV types varied across the studies, the majority were detected as part of a multiple infection (rather than as single infections), except in the Ludwig-McGill study. Subject characteristics that were commonly associated with multiple HPV infection included younger age and higher number of sexual partners (Table 2). CCCaST participants who reported condom use (“ever” versus “never”) and who were widowed/divorced were at higher risk of being infected with multiple HPV types, whereas subjects from the BCCR study who were married/common-law were at significantly lower risk compared with single individuals. Former smoking status was also associated with greater risk of multiple infections in HITCH and CCCaST studies, but not in the others.

**Table 2:** Characteristics of female participants at baseline/enrollment from five epidemiological studies, stratified by HPV status ^a^.

Characteristic	Ludwig-McGill study			McGill-Concordia study			HITCH study			CCCaST study			BCCR study		
	n = 2462			n = 636			n = 452			n = 10154			n = 981		
	S	M	OR	S	M	OR	S	M	OR	S	M	OR	S	M	OR
	n (%)	n (%)	(95% CI) ^b^	n (%)	n (%)	(95% CI) ^b^	n (%)	n (%)	(95% CI) ^b^	n (%)	n (%)	(95% CI) ^b^	n (%)	n (%)	(95% CI) ^b^
**Age**, years, mean (SD) ^c^	32.1	29.6	0.96	22.9	21.8	0.92	21.0	21.3	1.02	40.0	38.3	0.96	29.2	26.0	0.94
	(8.7)	(8.8)	(.94-.98)	(4.0)	(3.1)	(.87-.98)	(2.0)	(2.3)	(.92–1.14)	(8.3)	(7.1)	(.92-.99)	(7.6)	(7.1)	(.90-.98)
**Marital status**															
Single	106	72	1.00	118	158	1.00	111	187	1.00	90	86	1.00	95	103	1.00
	(12.7)	-22.3		(76.1)	(90.8)		(84.1)	(85.8)		(27.3)	(31.6)		(54.9)	(73.1)	
Married/common law	653	221	0.60	33	14	0.96	19	29	0.84	170	94	0.94	70	35	0.51
	(77.9)	(68.4)	(.34–1.09)	(21.3)	(8.0)	(.26–3.56)	(14.4)	(13.3)	(.44–1.63)	(51.7)	(34.6)	(.52–1.71)	(40.5)	(24.8)	(.30-.89)
Widowed/divorced	79	30	1.14	4	2	1.68	2	2	0.49	69	92	2.98	8	3	0.52
	(9.4)	(9.3)	(.62–2.11)	(2.6)	(1.2)	(.46–6.08)	(1.5)	(0.9)	(.03–8.33)	(21.0)	(33.8)	(1.48–6.01)	(4.6)	(2.1)	(.12–2.37)
**Age at sexual debut**															
< 16	165	61	1.00	36	44	1.00	37	67	1.00	31	28	1.00	44	52	1.00
	(19.9)	(19.2)		(25.7)	(26.8)		(28.5)	(30.7)		(15.7)	(20.4)		(26.4)	(38.2)	
≥ 16	665	257	1.31	104	120	1.09	93	151	1.14	166	109	0.7	123	84	0.84
	(80.1)	(80.8)	(.93–1.86)	(74.3)	(73.2)	(.67–1.78)	(71.5)	(69.3)	(.67–1.93)	(84.3)	(79.6)	(.37–1.31)	(73.6)	(61.8)	(.48–1.45)
**Lifetime # of sex partners**															
0–1	320	119	1.00	21	18	1.00	10	5	1.00	8	5	1.00	5	1	1.00
	(38.1)	(36.8)		(13.6)	(10.4)		(7.6)	(2.3)		(4.1)	(3.7)		(2.9)	(0.7)	
2–4	382	170	1.16	49	56	2.77	35	44	2.69	44	21	0.59	40	25	2.56
	(45.5)	(52.6)	(.82–1.66)	(31.8)	(32.4)	(1.64–4.66)	(26.5)	(20.2)	(.80–9.02)	(22.3)	(15.3)	(.16–2.24)	(23.4)	(17.7)	(.24–17.65)
≥ 5	137	34	1.41	84	99	6.71	87	169	3.88	145	111	0.95	126	115	4.23
	(16.3)	(10.5)	(.97–2.07)	(54.6)	(57.2)	(3.73–12.07)	(65.9)	(77.5)	(1.20–12.57)	(73.6)	(81.0)	(.27–3.28)	(73.7)	(81.6)	(.40–25.07)
**# of pregnancies**															
0	19	7	1.00	127	147	1.00	117	193	1.00	47	41	1.00	99	76	1.00
	(2.3)	(2.2)		(81.4)	(84.5)		(88.6)	(88.9)		(23.9)	(29.9)		(57.6)	(53.9)	
1–2	313	134	1.27	27	25	0.81	13	24	1.05	92	56	0.72	51	51	1.36
	(37.5)	(42.0)	(.49–3.30)	(17.3)	(14.4)	(.46–1.42)	(9.9)	(11.1)	(.48–2.29)	(46.7)	(40.9)	(.40–1.30)	(29.6)	(36.2)	(0.79–2.10)
≥ 3	503	178	1.3	2	2	1.09	2	0	N/E	58	40	0.79	22	14	0.88
	(60.2)	(55.8)	(.49–3.41)	(1.3)	(1.1)	(.17–7.13)	(1.5)	(0.0)	N/E	(29.4)	(29.2)	(.41–1.51)	(12.8)	(9.9)	(0.42–1.84)
**OC use**															
Never	717	260	1.00	30	39	1.00	20	31	1.00	122	106	1.00	11	5	1.00
	(85.5)	(80.5)		(20)	(22.9)		(15.4)	(14.2)		(36.9)	(38.7)		(6.4)	(3.6)	
Ever ^d^	122	63	0.92	120	131	0.95	110	187	0.91	87	66	1.45	161	135	1.26
	(14.5)	(19.5)	(.61–1.39)	(80.0)	(77.1)	(.60–1.50)	(84.6)	(85.8)	(.46–1.79)	(26.3)	(24.1)	(.66–3.20)	(93.6)	(96.4)	(.34–4.66)
Missing ^e^	-	-	-	-	-	-	-	-	-	122	102	1.11	-	-	-
	-	-	-	-	-	-	-	-	-	(36.8)	(37.2)	(.44–2.78)	-	-	-
**Condom use**															
Never	320	105	1.00	7	7	1.00	4	2	1.00	159	119	1.00	9	5	1.00
	(38.8)	(32.7)		(4.6)	(4.1)		(3.1)	(0.9)		(48.0)	(43.4)		(5.2)	(3.6)	
Rarely/sometimes/	472	198	1.22	84	105	0.7	46	86	0.62	55	60	2.53	62	54	1.6
ever ^d^	(57.2)	(61.7)	(.91–1.63)	(55.6)	(61.4)	(.30–1.66)	(35.1)	(39.4)	(.08.26)	(16.6)	(21.9)	(1.15.54)	(36.0)	(38.6)	(.45.66)
Regularly/always	33	18	1.39	60	59	0.99	81	130	0.5	-	-	-	101	81	0.86
	(4)	(5.6)	(.74–2.64)	(39.7)	(34.5)	(.41–2.38)	(61.8)	(59.6)	(.04–6.60)	-	-	-	(58.7)	(57.8)	(.52–1.43)
Missing ^e^	-	-	-	-	-	-	-	-	-	117	95	1.77	-	-	-
	-	-	-	-	-	-	-	-	-	(35.4)	(34.7)	(.56–5.56)	-	-	-
**Cigarette smoking**															
Never smoker	386	155	1.00	96	96	1.00	85	114	1.00	49	150	1.00	78	56	1.00
	(46.0)	(48.0)		(61.9)	(55.5)		(64.4)	(52.3)		(64.5)	(53.6)		(45.1)	(39.7)	
Former smoker	145	46	0.91	35	45	1.36	29	76	1.99	19	90	1.94	39	25	0.8
	(17.3)	(14.24)	(.67–1.22)	(22.6)	(26.0)	(.83–2.24)	(22.0)	(34.9)	(1.13–3.49)	(25.0)	(32.1)	(1.10–3.42)	(22.5)	(17.7)	(.41–1.56)
Current smoker	308	122	0.74	24	32	1.12	18	28	0.97	8	40	1.85	56	60	1.06
	(36.7)	(37.8)	(.50–1.11)	(15.5)	(18.5)	(.67–1.88)	(13.6)	(12.8)	(.49–1.94)	(10.5)	(14.3)	(.96–3.56)	(32.4)	(42.6)	(.61–1.85)

Abbreviations: CI, confidence interval; HPV, human papillomavirus; S, single HPV infection; M, multiple HPV infection; N, number; N/E, not able to estimate; OC, oral contraceptive; OR, odds ratio; Ref, reference; SD, standard deviation.

^a^ Subject was assigned to multiple HPV infection category if concurrent HPV co-infection was observed at any clinic visit (baseline or follow-up).

^b^ Odds ratios were adjusted for all variables listed in the table.

^c^ Age was modeled as a linear variable with 1 degree-of-freedom.

^d^ Checklist was used in CCCaST to evaluate whether subjects “ever” used OCs or condoms, along with other contraceptive methods.

^e^ For CCCaST only, “missing” was included in analysis for OC and condom use variables

**Fig 6:**
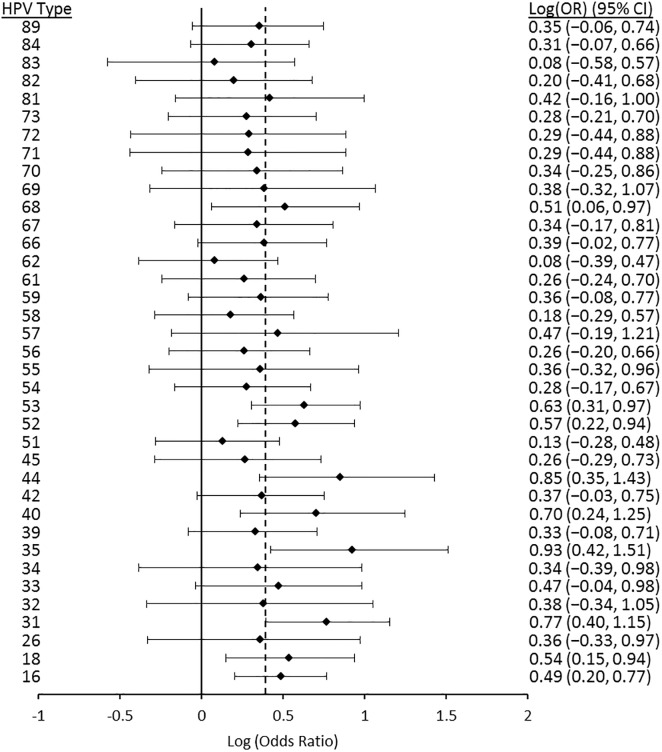
Log (odds ratios) and 95% confidence intervals for HPVs 6/11 for co-infection with other HPV types. Estimates were obtained from logistic regression models adjusted for all other types, age, lifetime number of sexual partners, and study. The dashed line represents the average pooled log(OR) from hierarchical logistic regression, which was 0.39 (95%CI: 0.24–0.53). The analysis included pooled results from Ludwig-McGill, McGill-Concordia, HITCH, BCCR, and CCCaST studies. Approximately half of subjects from CCCaST (n = 5754; St. John’s site) were excluded from these analyses due to missing information regarding lifetime number of sexual partners.

**Fig 7:**
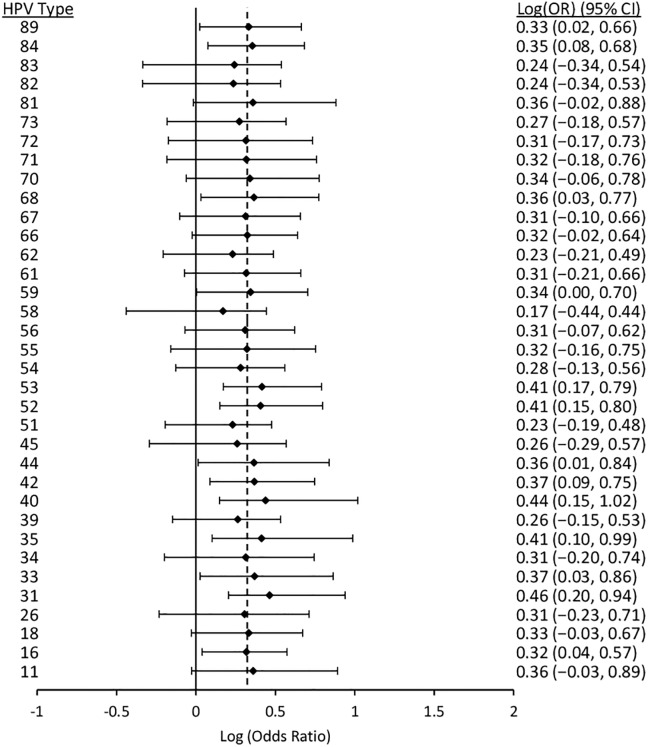
Log (odds ratios) and 95% confidence intervals for HPV6 for co-infection with other HPV types. Estimates were obtained from logistic regression models adjusted for all other types, age, lifetime number of sexual partners, and study. The dashed line represents the average pooled log(OR) from hierarchical logistic regression, which was 0.32 (95%CI: 0.20–0.43). The analysis included pooled results from McGill-Concordia, HITCH, BCCR, and CCCaST studies. Approximately half of subjects from CCCaST (n = 5754; St. John’s site) were excluded from these analyses due to missing information regarding lifetime number of sexual partners.

**Fig 8:**
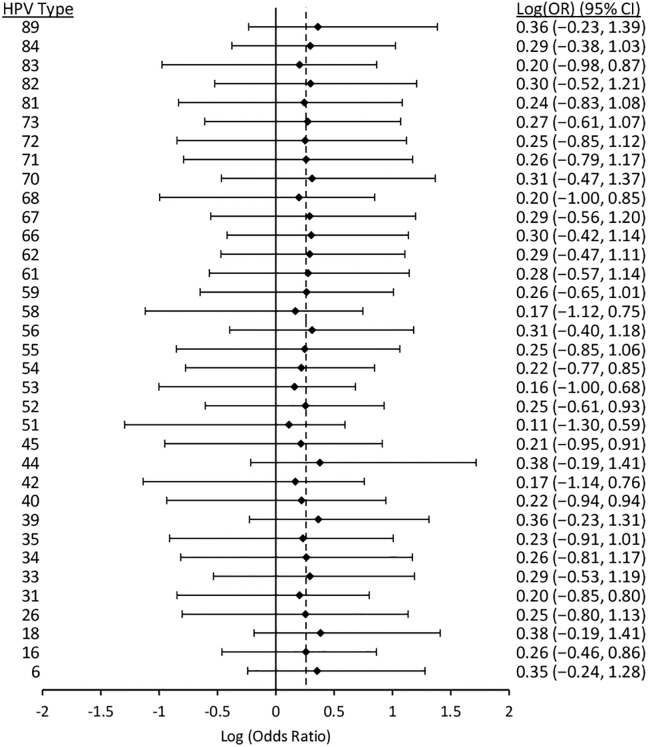
Log (odds ratios) and 95% confidence intervals for HPV11 for co-infection with other HPV types. Estimates were obtained from logistic regression models adjusted for all other types, age, lifetime number of sexual partners, and study. The dashed line represents the average pooled log(OR) from hierarchical logistic regression, which was 0.26 (95%CI: -0.07–0.50). The analysis included pooled results from McGill-Concordia, HITCH, BCCR, and CCCaST studies. Approximately half of subjects from CCCaST (n = 5754; St. John’s site) were excluded from these analyses due to missing information regarding lifetime number of sexual partners.

**Fig 9:**
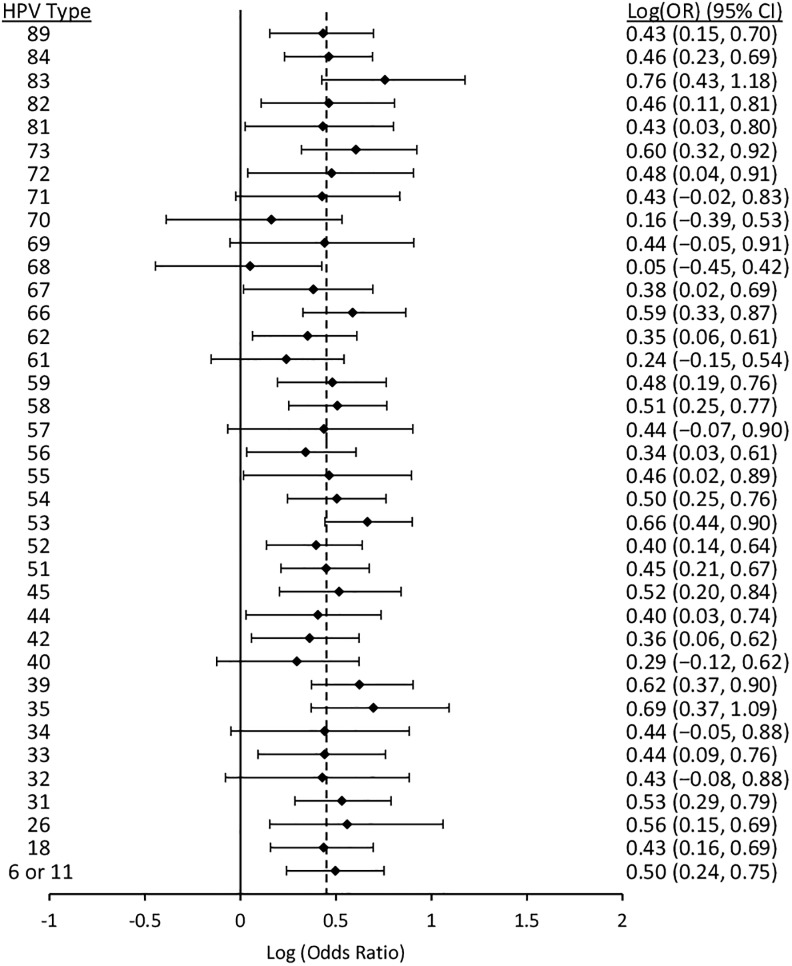
Log (odds ratios) and 95% confidence intervals for HPV16 for co-infection with other HPV types. Estimates were obtained from logistic regression models adjusted for all other types, age, lifetime number of sexual partners, and study. The dashed lines represents the average pooled log(OR) from hierarchical logistic regression, which was 0.45 (95%CI: 0.34–0.55). The analysis included pooled results from Ludwig-McGill, McGill-Concordia, HITCH, BCCR, and CCCaST studies. Approximately half of subjects from CCCaST (n = 5754; St. John’s site) were excluded from these analyses due to missing information regarding lifetime number of sexual partners.

**Fig 10:**
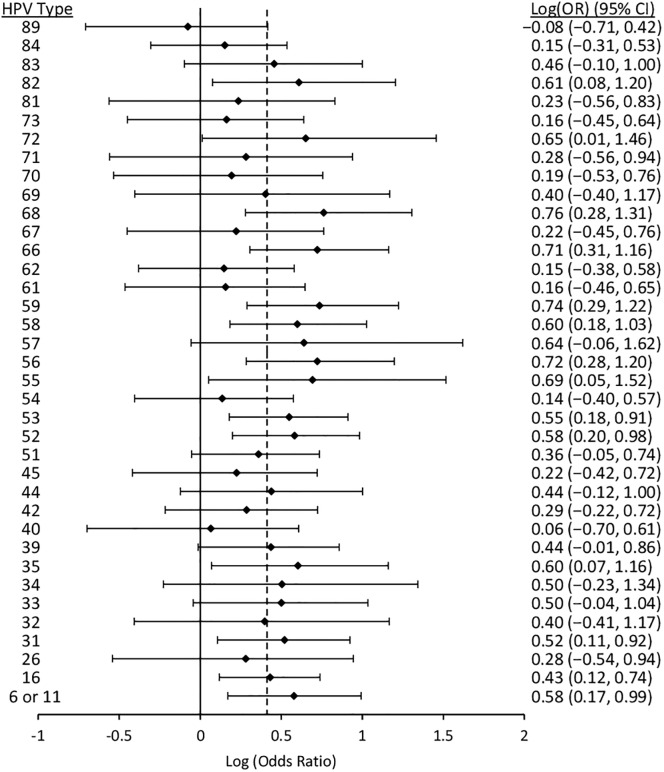
Log (odds ratios) and 95% confidence intervals for HPV18 for co-infection with other HPV types. Estimates were obtained from logistic regression models adjusted for all other types, age, lifetime number of sexual partners, and study. The dashed line represents the average pooled log(OR) from hierarchical logistic regression, which was 0.41 (95%CI: 0.23–0.57). The analysis included pooled results from Ludwig-McGill, McGill-Concordia, HITCH, BCCR, and CCCaST studies. Approximately half of subjects from CCCaST (n = 5754; St. John’s site) were excluded from these analyses due to missing information regarding lifetime number of sexual partners.

Figs 6 to 10 display results from the logistic regression models. Each of the graphs present OR estimates for type-type associations on the natural log scale; therefore, (log)OR estimates greater than zero correspond to ORs greater than one (i.e., positive associations between HPV types), and the opposite for (log)OR estimates below zero. In our pooled regression analyses (including data from all five studies), no statistically significant negative associations were observed between vaccine-targeted HPV types (HPVs 6, 11, 16, and 18) and any other types (Figs 6, 7, 8, 9 and 10). In fact, the only point estimate indicating a negative association observed was between HPV18 and 89 (OR = 0.92, 95%CI: 0.49–1.52); however, there was insufficient precision to reject the null hypothesis of no association. These analyses included adjustment for other HPV types, age and lifetime number of sexual partners, but excluded over half of CCCaST study participants (n = 5754) due to missing sexual history information from St. John’s study site participants. In our analyses adjusted for other HPV types and age only (including all CCCaST subjects), results were similar, i.e., no negative associations were observed, and OR estimates were generally higher (S1 Fig).

Across the studies with individual typing information for HPVs 6 and 11 (i.e., all other than Ludwig-McGill study), HPV11 was detected in only 23 of 16027 specimens. In our analyses of HPVs 6 and 11 separately (Figs 7 and 8; S1 Fig, panels B and C) and grouped together (Fig 6; S1 Fig, panel A), results were similar between HPVs 6/11 and HPV6, but not between HPVs 6/11 and HPV11. In our fully adjusted pooled analyses (Figs 6, 7, 8, 9 and 10), many statistically significant positive associations (ORs>1.0, 95% CIs excluded 1.0) were observed between HPVs 6/11 and other types (HPVs 68, 53, 52, 44, 40, 35, 31, 18, and 16), as well as between HPV6 and other types (HPVs 89, 84, 68, 53, 52, 44, 42, 35, 33, 31, and 16); however, no significant positive associations were observed involving HPV11. HPV16 was positively associated with all except for the following HPV types: 71, 70, 69, 68, 61, 57, 40, 34, and 32. Finally, HPV18 was positively associated with HPVs 82, 72, 68, 66, 59, 58, 56, 55, 53, 52, 35, 31, 16, 6/11. In summary, significant positive associations were observed involving one or more vaccine-targeted HPV types, with all except for seven other types (HPVs 71, 70, 69, 61, 57, 34 and 31). In our pooled analyses not controlling for lifetime number of sexual partners (S1 Fig; all CCCaST specimens included), all of the HPV types listed above remained statistically significant in each of the respective analyses; and also included additional significant types (but all with ORs>1.0).

In our fully adjusted pooled analyses focusing on HPVs 6/11, 6, 11, 16 and 18 (Figs 6, 7, 8, 9 and 10), the average pooled (log)ORs for co-infections involving these HPV types estimates (i.e., the value that individual type-type associations were “shrunk” towards in each of the respective analyses) was 0.39 (95%CI: 0.24–0.53), 0.32 (95%CI: 0.20–0.43), 0.26 (95%CI: -0.07–0.50), 0.45 (95%CI: 0.34–0.55), and 0.41 (95%CI: 0.23–0.57), respectively. The average pooled ORs for co-infections involving vaccine-targeted HPV types with other types varied across the five studies; however, no consistent trend of higher or lower pooled ORs was observed for any of the studies (S2, S3, S4, S5 and S6 Figs). Because very few HPV11 infections were observed in the BCCR and CCCaST studies (n = 2 and n = 1, respectively), individual study results for this vaccine-target type were only presented for the McGill-Concordia and HITCH studies (S4 Fig).

## Discussion

Assessment of pre-vaccine epidemiological data can provide insights concerning natural HPV type competition and the potential for type replacement [10]. HPV types that naturally compete with HPVs 6, 11, 16, and/or 18 may be more likely to fill the ecological niches vacated by these vaccine-target types. The US Food and Drug Administration and Health Canada recently approved Merck’s new HPV vaccine (Gardasil 9©) that protects against the same four HPV types as the original Gardasil vaccine (6, 11, 16, and 18), plus additional oncogenic HPV types 31, 33, 45, 52, and 58 [38]. However, despite the availability of this new nonavalent vaccine, concern about type replacement remains important. Millions of women have already been vaccinated using either the bivalent or quadrivalent formulations, and both first-generation vaccines continue to be administered in many countries.

In general, our results support previous studies, which mainly reported null or positive associations between different HPV types [18–26]. Recently, Vaccarella and colleagues used a number of large data sets to evaluate clustering patterns between HPV types (via hierarchical regression models with woman-level random effects), identifying few negative associations and some positive associations, which they generally attributed to diagnostic artifacts [21–24]. Similarly, Chaturvedi and colleagues reported very few negative or positive associations in examining HPV co-infection patterns among women from the Costa Rica Vaccine Trial, concluding that HPV infections seemed to occur independently in this population [19]. Furthermore, in a recent pooled analysis, including information from three diverse study populations in the Netherlands, Mollers and colleagues also reported no significant pairwise interactions, but did suggest that clustering patterns differed across risk groups and across types, particularly between low- and high-risk HPV types [25]. In general, phylogenetic relatedness did not strongly influence clustering patterns in these prior studies; whereas in our study, HPV16 (α-9) was positivity associated with all related types, and HPVs 6/11 and 18 were positively associated with related types 44 (α-10) and 59 (α-7), respectively.

Across the five studies, there were more than 38,000 cervical specimens with valid HPV testing results, which makes the current pooled analysis one of the largest studies on this topic to date. As a result, we were able to evaluate associations between vaccine-targeted HPV types with all others, including rare types. The application of Bayesian methods incorporating shrinkage further improved our precision, and still allowed us to adjust for all relevant covariates and presence of other HPV types in our models. However, any improvement in precision resulting from shrinkage comes at the expense of introducing some bias [37]. To explore if our results may have been meaningfully different according to traditional analytic methods (i.e., without this bias/precision trade-off), we performed sensitivity analyses using maximum likelihood estimation. As expected, this approach led to wider confidence intervals, but importantly it did not lead to any statistically significant ORs less than one (data not shown). Although we did not observe any statistically significant negative associations in our study, we did observe a large number of positive associations. We suspect that most significant positive associations may be attributed to residual confounding, i.e., due to our inability to control for all risk factors of multiple-type HPV infections (e.g., host susceptibility, immunological differences, or other unmeasured behaviour risk factors). For example, in our analyses including all CCCaST specimens (i.e., unadjusted for sexual history), confounding may explain the higher OR estimates and greater number of HPV types found to be positively associated with HPVs 6, 11, 16 and 18. To ensure that analyses of type interactions are focused among those with sufficient HPV exposure opportunity, we and others have previously explored the effect of restricting the study sample to individuals with ≥1 HPV infection [18, 39–41]. However, albeit insightful, this approach leads to a form of selection bias, referred to as collider stratification bias [42], and was therefore not applied in the current study.

Despite variation in key demographic and behavioural risk factors across individual studies, results were generally consistent after adjustment for age, lifetime number of sexual partners, and other HPV types. Nonetheless, it is important to consider how differences in important HPV risk factors may have impacted our results and ability to pool information. For example, in the HITCH and McGill-Concordia studies, participants were younger than those in the other studies and therefore we may suspect that infections in these two studies are more likely to represent incident or recently acquired infections rather than persistent infections. This may have important implications since oncogenic vaccine-targeted types, such as HPVs 16 and 18, are more likely to be persistent and detected with other HPV types, leading to higher OR estimates in the current study. In a separate recent analysis conducted to evaluate incidence and clearance of individual HPV types according to infection with vaccine-targeted HPV types, we observed similar two-year incidence rates (any infection) in the Ludwig-McGill, McGill-Concordia, and HITCH cohort studies (23.6%, 27.0%, and 18.3%, respectively) [15]. Also, compared with their younger counterparts in the McGill-Concordia and HITCH studies, Ludwig-McGill participants were more likely to clear their existing infections within two years [15]. These results suggest that infections observed across studies in the current analysis may represent a similar proportion of incident or recently acquired and persistent infections. Importantly, results from this cohort analysis also did not provide any evidence of HPV type competition, i.e., individuals with vaccine-targeted HPV types were not less likely to acquire other types or more likely to clear their existing infections [15].

The five studies from which specimens were collected all utilized broad spectrum PCR assays to test for the presence of HPV. Although these assays are able to amplify and detect a large number of HPV genotypes and may detect as few as 10 copies of viral DNA for most common genital HPV types [32, 43, 44], previously we discussed concerns regarding the sensitivity of consensus assays in the context of type replacement evaluation, particularly in situations where specimens are coinfected with multiple HPV types [10]. In a recent analysis conducted to evaluate possible “masking” of HPV52 in the presence of HPV16, we observed a significant positive association between HPV16 viral load and masking of HPV52 [45]. Other PCR assays have been developed with reported high sensitivity for detection of multiple HPV types from coinfected specimens, e.g., using array primer extension (APEX) for typing [46]; however, these methods remain less common. In addition, there is also the possibility that assay specificity may be reduced as a consequence of probe cross-reactivity [47], which may explain the tendency for some phylogenetically related types to cluster together. However, considering that most HPV types from the α-9 species are also classified as definite carcinogens by the International Agency for Research on Cancer (all except for HPV67) [48], they are also more likely to persist (than low-risk types) and therefore are more likely to be detected together with other types [25]. The observation that certain HPV types (e.g., HPV16) were consistently observed more frequently than others across individual studies suggests that a competitive advantage exists for some HPV types.

Previous cross-sectional and cohort studies focusing on different populations and employing unique analytic/genotyping methods have failed to provide consistent or strong evidence that negative pairwise HPV interactions exist [12–26]. The current study adds to this literature by providing additional reassurance that—owing to the lack of HPV type competition—type replacement appears unlikely. Since we did not include females who received prophylactic HPV vaccines for comparison in this study, we must assume that no major differences in acquiring other types exist among females who are naturally uninfected with vaccine-target types. Eventually, a definitive answer to this question of whether HPV type replacement has occurred will come from long-term surveillance studies which compare pre- and post-vaccination type-specific HPV prevalence rates, and which properly account for possible diagnostic artifacts [10, 45].

## Supporting Information

S1 Fig Log (odds ratios) and 95% confidence intervals for HPVs 6/11, 6, 11, 16 and 18 for co-infection with other HPV types (panels A-E, respecively). Estimates were obtained from logistic regression models adjusted for all other HPV types, and age only. In panels A-E, the dashed lines represent the average pooled log(OR) from hierarchical logistic regression, which were 0.43 (95%CI: 0.30–0.56), 0.38 (95%CI: 0.28–0.48), 0.26 (95%CI: -0.02–0.56), 0.51 (95%CI: 0.41–0.60), and 0.47 (95%CI: 0.33–0.60), respectively. All analyses included pooled results from Ludwig-McGill (except for panels B and C; due to our inability to distinguish between HPVs 6 and11), McGill-Concordia, HITCH, BCCR, and CCCaST studies.
(TIF)

S2 Fig Log (odds ratios) and 95% confidence intervals for HPV6/11 with other HPV types from the Ludwig-McGill, McGill-Concordia, HITCH, BCCR, and CCCaST studies (panels A-E, respecively). Estimates were obtained from logistic regression models adjusted for all other HPV types, age, and lifetime number of sexual partners (except CCCaST; adjusted for other HPV types and age only). In panels A-E, the dashed lines represent the average pooled log(OR) from hierarchical logistic regression, which were 0.61 (95%CI: 0.18–0.88), 0.19 (95%CI: -0.31–0.51), 0.27 (95%CI: 0.08–0.45), 0.96 (95%CI: 0.54–1.39), and 0.50 (95%CI: -0.30–1.088), respectively.
(TIF)

S3 Fig Log (odds ratios) and 95% confidence intervals for HPV6 with other HPV types from the McGill-Concordia, HITCH, BCCR, and CCCaST studies (panels A-D, respecively). Estimates were obtained from logistic regression models adjusted for all other HPV types, age, and lifetime number of sexual partners (except CCCaST; adjusted for other HPV types and age only). In panels A-D, the dashed lines represent the average pooled log(OR) from hierarchical logistic regression, which were 0.22 (95%CI: -0.30–0.56), 0.26 (95%CI: 0.07–0.41), 0.26 (95%CI: -0.02–0.56), 0.54 (95%CI: 0.14–0.91), and 0.84 (95%CI: 0.21.18), respectively.
(TIF)

S4 Fig Log (odds ratios) and 95% confidence intervals for HPV11 with other HPV types from the McGill-Concordia and HITCH studies (panels A and B, respecively). Estimates were obtained from logistic regression models adjusted for all other HPV types, age, and lifetime number of sexual partners. In panels A and B, the dashed lines represent the average pooled log(OR) from hierarchical logistic regression, which were -0.19 (95%CI:-1.42–0.52) and 0.21 (95%CI: -0.48–0.60), respectively.
(TIF)

S5 Fig Log (odds ratios) and 95% confidence intervals for HPV16 with other HPV types from the Ludwig-McGill, McGill-Concordia, HITCH, BCCR, and CCCaST studies (panels A-E, respecively). Estimates were obtained from logistic regression models adjusted for all other HPV types, age, and lifetime number of sexual partners (except CCCaST; adjusted for other HPV types and age only). In panels A-E, the dashed lines represent the average pooled log(OR) from hierarchical logistic regression, which were 0.53 (95%CI: 0.21–0.77), 0.43 (95%CI: 0.25–0.60), 0.32 (95%CI: 0.22–0.42), 0.12 (95%CI: -0.47–0.46), and 0.70 (95%CI: 0.47–0.88), respectively.
(TIF)

S6 Fig Log (odds ratios) and 95% confidence intervals for HPV18 with other HPV types from the Ludwig-McGill, McGill-Concordia, HITCH, BCCR, and CCCaST studies (panels A-E, respecively). Estimates were obtained from logistic regression models adjusted for all other HPV types, age, and lifetime number of sexual partners (except CCCaST; adjusted for other HPV types and age only). In panels A-E, the dashed lines represent the average pooled log(OR) from hierarchical logistic regression, which were 0.64 (95%CI: 0.33–0.84), 0.38 (95%CI: -0.10–0.71), 0.30 (95%CI: -0.01–0.59), -0.63 (95%CI: -3.41–0.47), and 0.50 (95%CI: -0.30–1.088), respectively.
(TIF)

## Abbreviations

CI: confidence interval
HPV: human papillomavirus
HR-HPV: high-oncogenic risk human papillomavirus
LR-HPV: low-oncogenic risk human papillomavirus
OR: odds ratio
PCR: polymerase chain reaction
