# Supplementary figures and images for: Epidemiologic Evaluation of Human Papillomavirus Type Competition and the Potential for Type Replacement Post-Vaccination
Source: PLoS One. 2016 Dec 22;11(12):e0166329. doi: 10.1371/journal.pone.0166329 (PMC5178990; doi:10.1371/journal.pone.0166329)

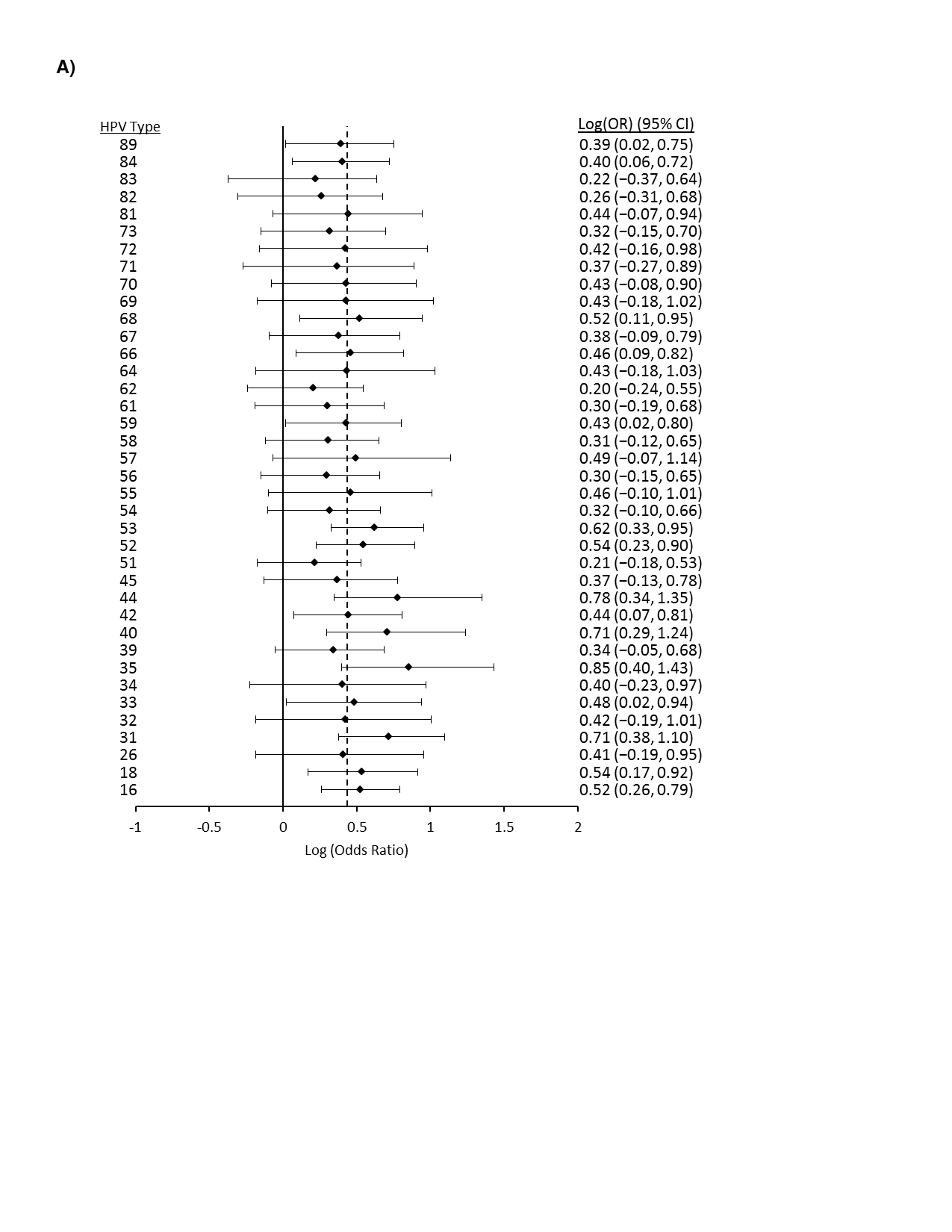

Supplement: S1 Fig — Estimates were obtained from logistic regression models adjusted for all other HPV types, and age only. In panels A-E, the dashed lines represent the average pooled log(OR) from hierarchical logistic regression, which were 0.43 (95%CI: 0.30–0.56), 0.38 (95%CI: 0.28–0.48), 0.26 (95%CI: -0.02–0.56), 0.51 (95%CI: 0.41–0.60), and 0.47 (95%CI: 0.33–0.60), respectively. All analyses included pooled results from Ludwig-McGill (except for panels B and C; due to our inability to distinguish between HPVs 6 and11), McGill-Concordia, HITCH, BCCR, and CCCaST studies. (TIF) [file pone.0166329.s001.tif]

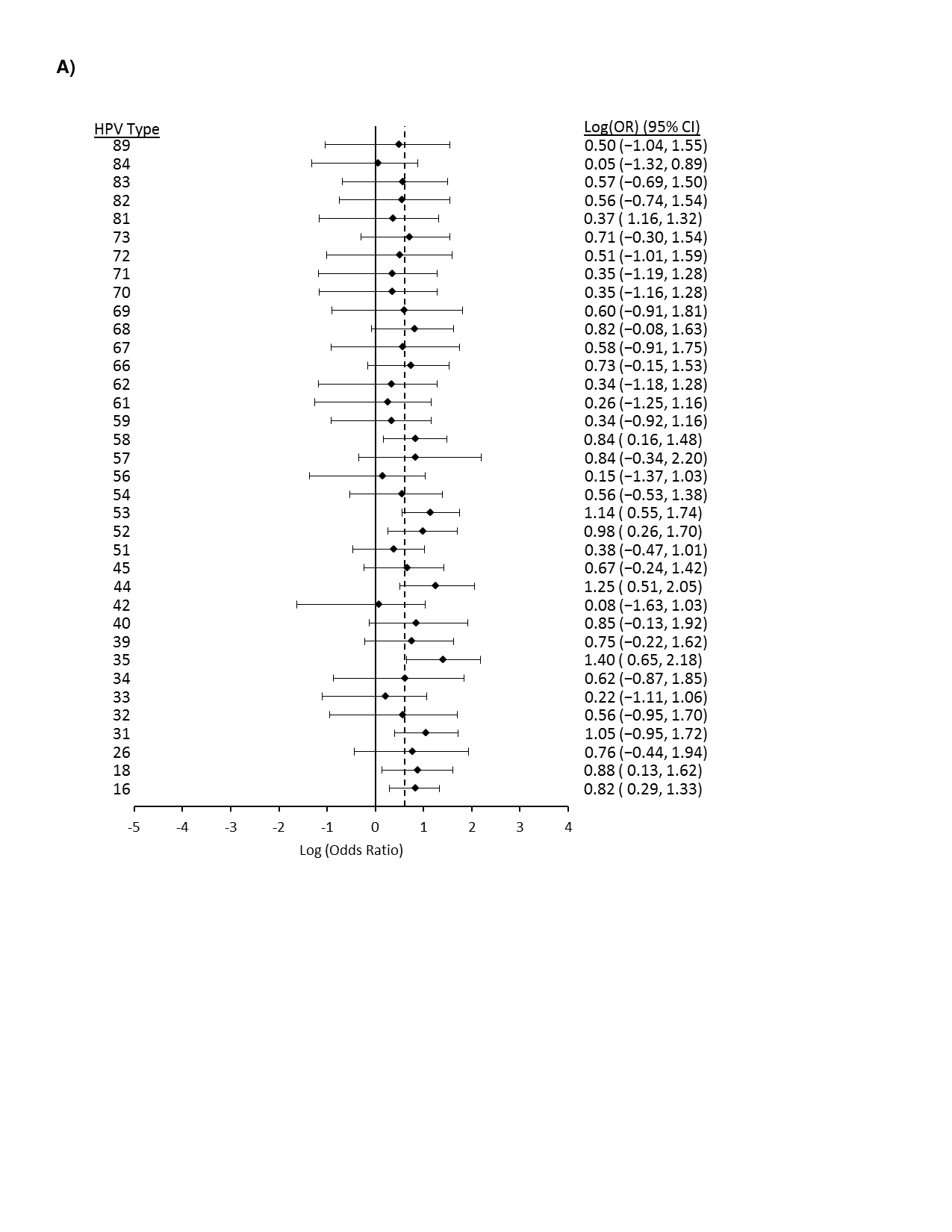

Supplement: S2 Fig — Estimates were obtained from logistic regression models adjusted for all other HPV types, age, and lifetime number of sexual partners (except CCCaST; adjusted for other HPV types and age only). In panels A-E, the dashed lines represent the average pooled log(OR) from hierarchical logistic regression, which were 0.61 (95%CI: 0.18–0.88), 0.19 (95%CI: -0.31–0.51), 0.27 (95%CI: 0.08–0.45), 0.96 (95%CI: 0.54–1.39), and 0.50 (95%CI: -0.30–1.088), respectively. (TIF) [file pone.0166329.s002.tif]

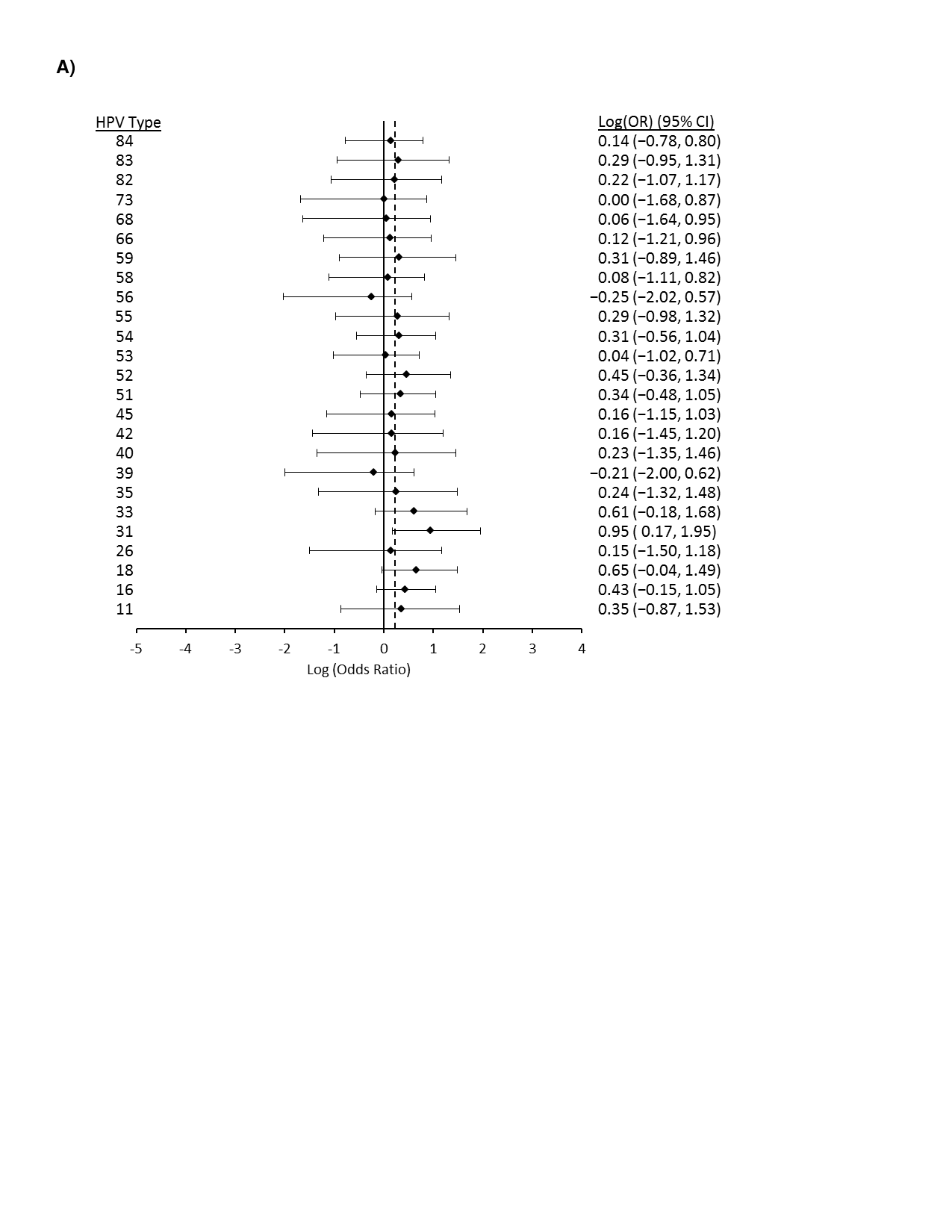

Supplement: S3 Fig — Estimates were obtained from logistic regression models adjusted for all other HPV types, age, and lifetime number of sexual partners (except CCCaST; adjusted for other HPV types and age only). In panels A-D, the dashed lines represent the average pooled log(OR) from hierarchical logistic regression, which were 0.22 (95%CI: -0.30–0.56), 0.26 (95%CI: 0.07–0.41), 0.26 (95%CI: -0.02–0.56), 0.54 (95%CI: 0.14–0.91), and 0.84 (95%CI: 0.21.18), respectively. (TIF) [file pone.0166329.s003.tif]

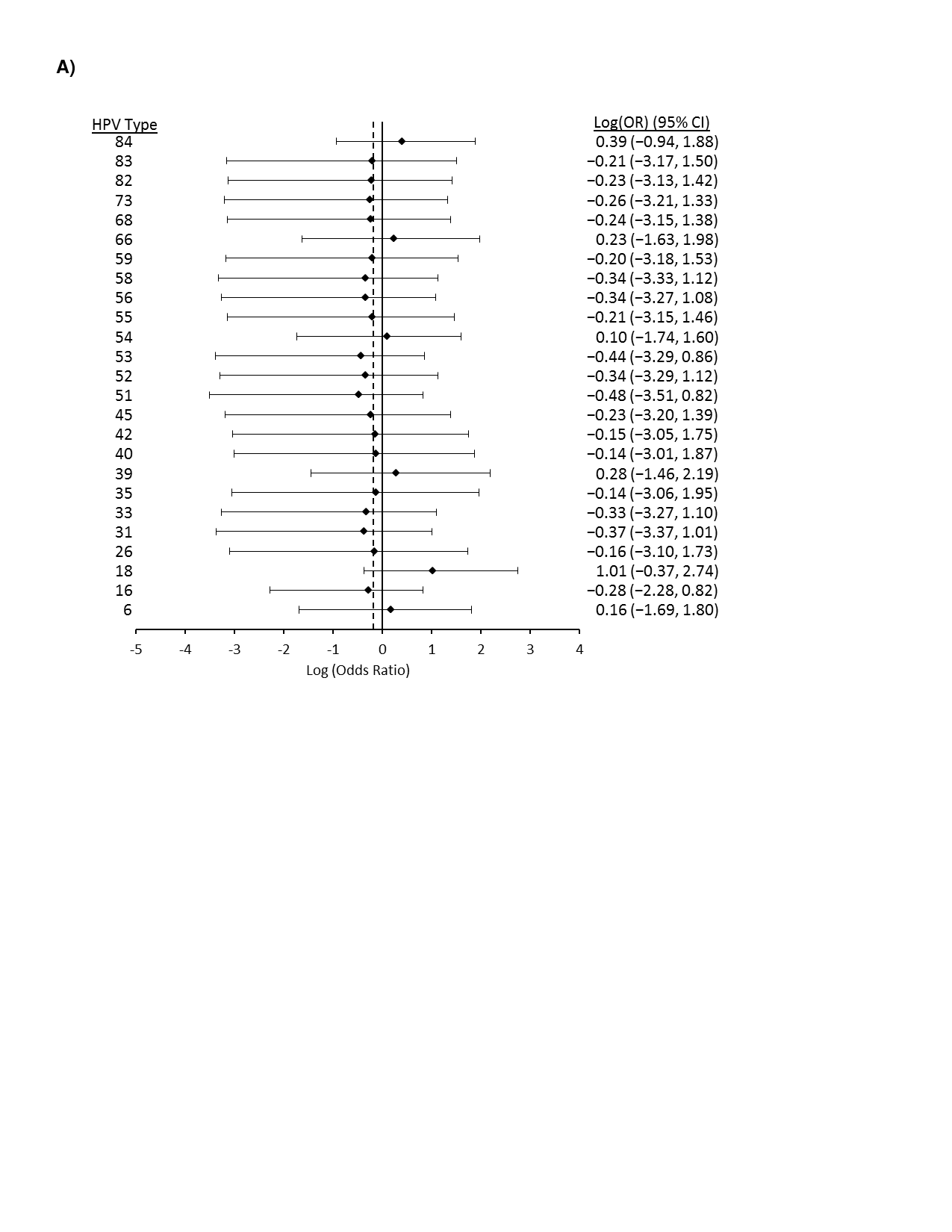

Supplement: S4 Fig — Estimates were obtained from logistic regression models adjusted for all other HPV types, age, and lifetime number of sexual partners. In panels A and B, the dashed lines represent the average pooled log(OR) from hierarchical logistic regression, which were -0.19 (95%CI:-1.42–0.52) and 0.21 (95%CI: -0.48–0.60), respectively. (TIF) [file pone.0166329.s004.tif]

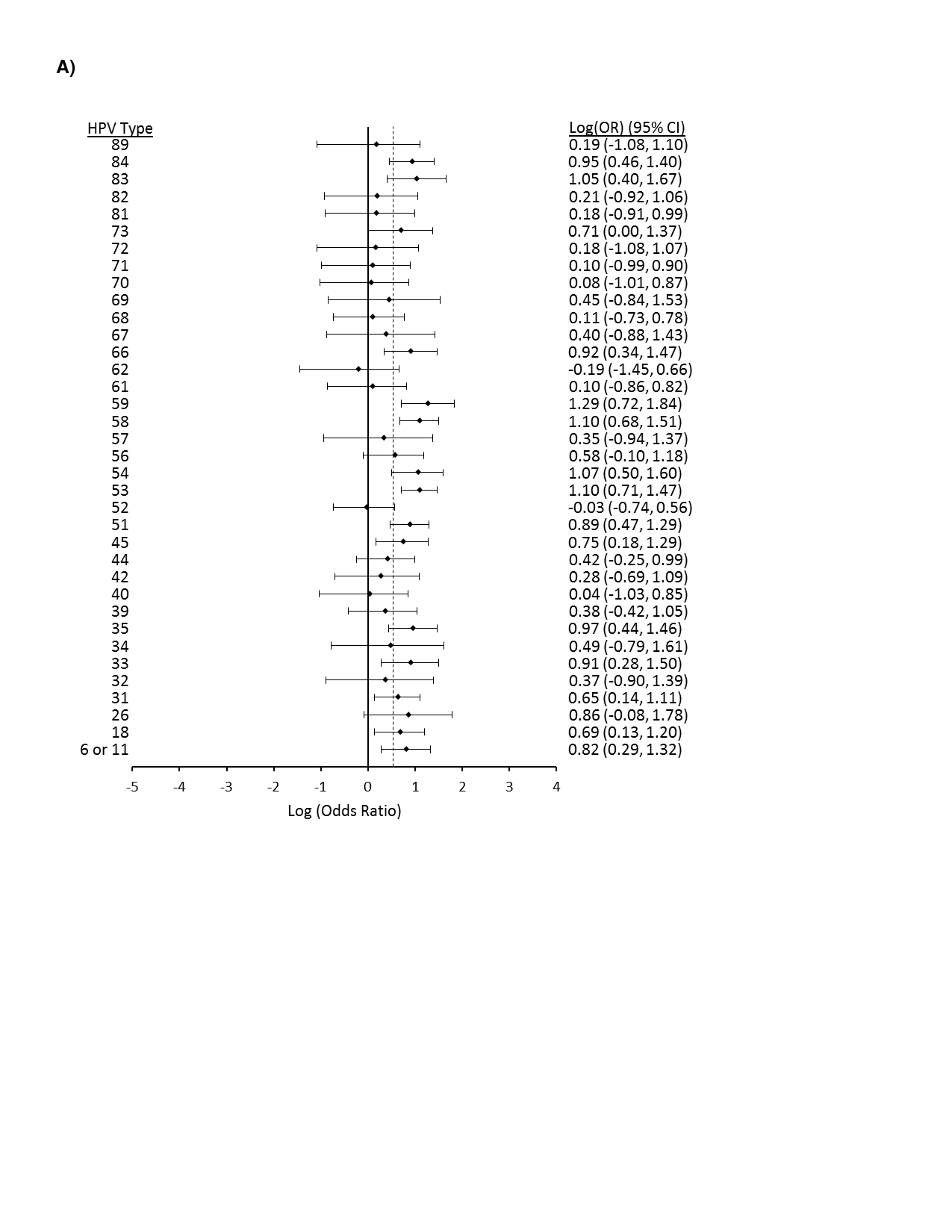

Supplement: S5 Fig — Estimates were obtained from logistic regression models adjusted for all other HPV types, age, and lifetime number of sexual partners (except CCCaST; adjusted for other HPV types and age only). In panels A-E, the dashed lines represent the average pooled log(OR) from hierarchical logistic regression, which were 0.53 (95%CI: 0.21–0.77), 0.43 (95%CI: 0.25–0.60), 0.32 (95%CI: 0.22–0.42), 0.12 (95%CI: -0.47–0.46), and 0.70 (95%CI: 0.47–0.88), respectively. (TIF) [file pone.0166329.s005.tif]

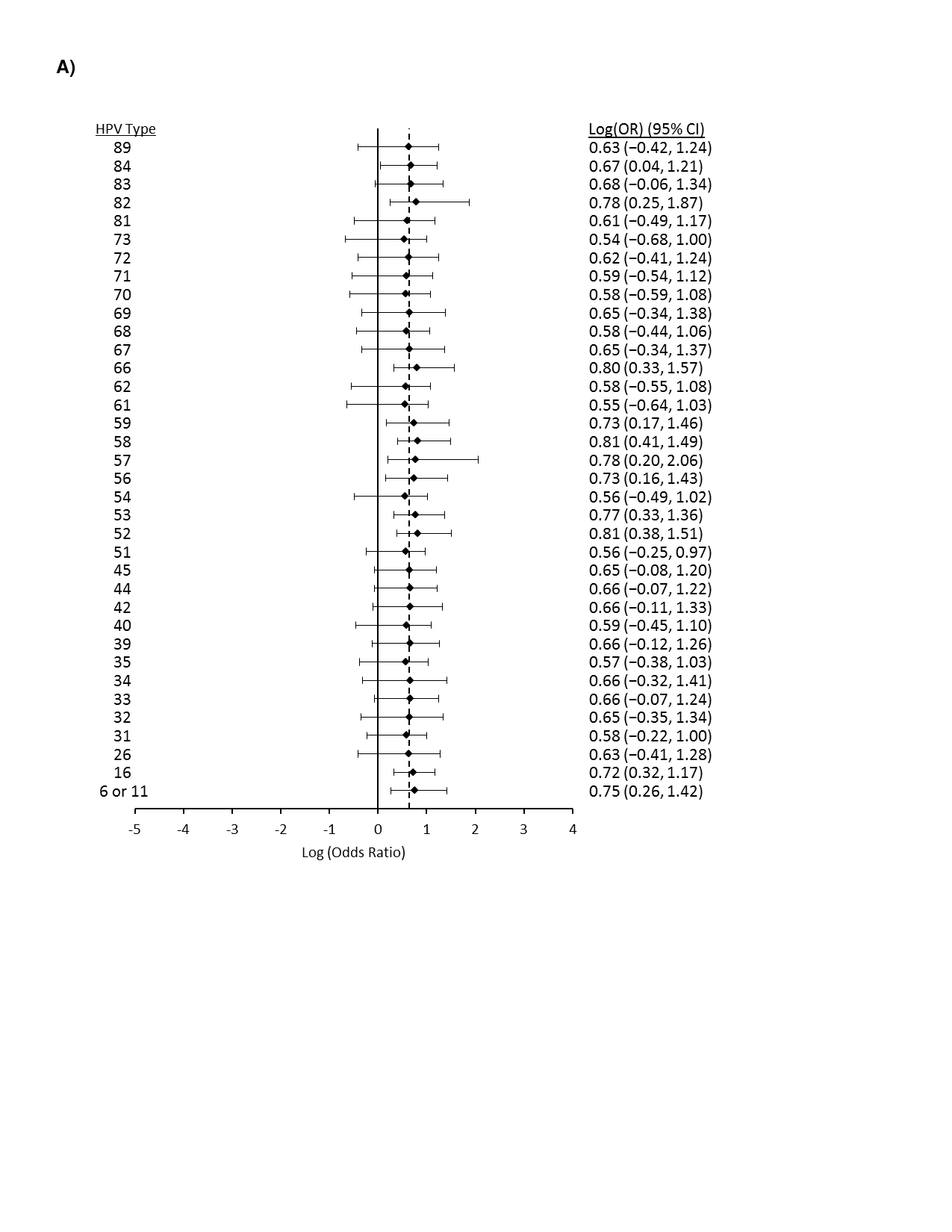

Supplement: S6 Fig — Estimates were obtained from logistic regression models adjusted for all other HPV types, age, and lifetime number of sexual partners (except CCCaST; adjusted for other HPV types and age only). In panels A-E, the dashed lines represent the average pooled log(OR) from hierarchical logistic regression, which were 0.64 (95%CI: 0.33–0.84), 0.38 (95%CI: -0.10–0.71), 0.30 (95%CI: -0.01–0.59), -0.63 (95%CI: -3.41–0.47), and 0.50 (95%CI: -0.30–1.088), respectively. (TIF) [file pone.0166329.s006.tif]
